# Central Ill. Med. Society—Report on “Minor Operations in Surgery”

**Published:** 1875-02

**Authors:** J. L. White

**Affiliations:** Bloomington


CENTRAL ILLINOIS MEDICAL SOCIETY.

### REPORT ON "MINOR OPERATIONS IN SURGERY."

(Read before the Society, at its meeting held at Gibson, Dec. 8, 1874.)

By J. L. WHITE, M.D., of Bloomington.

Since the last meeting of this Society no great event has transpired to make an era in the history of surgical science, and I find it difficult to offer you a report confined to the surgery of the past six months. I have therefore chosen rather to call your attention to the little things connected with the practice of surgery, or such of them as occur to my mind, hoping some little item may prove of importance to you all, and that the paper may elicit from you items of interest and importance to me. In the affairs of life, little things, trifling in themselves, many times hardly deemed worthy of notice, make up the sum to individuals of human happiness or misery. It is notorious that, many times, little trials and annoyances of daily occurrence, to which all are subject, are borne with less fortitude than some great misfortune, coming perhaps but once in a lifetime. So, on the other hand, as our earth is made up of little atoms, little items of knowlege—many of them so small as often to be deemed unworthy of attention—go to make up the expert in every department of the broad domain of science. In our medical colleges the great facts of surgery, both scientific and operative, are clearly set forth, and the principles upon which the science rests faithfully enunciated; but many of the minor details are left for each individual to discover for himself, as best he may, and there are but few surgeons who do not often gather little facts, not noticed by standard authors, a previous knowledge of which they realize would have been of advantage to them and their patients.

Among mechanics, one of the best evidences of the skilled workman is the manner and condition in which his tools are kept. "A place for everything and everything in its place," is a motto which should be ever present in the mind of a surgeon. To be able to lay your hands on any instrument wanted at any time, by night or day, or to tell any one just where in your office it may be found, is very desirable, and many times very important. To do this, your armentaria must be kept under lock and key. This is desirable for another reason. Instruments exposed to view and to reach are liable to be handled by curious, thoughtless observers, and are also more liable to become damp and to rust.

When an operation is to be performed, every instrument, ligature or appliance that may possibly be required should be spread out in such a way that they may be within easy reach and in perfect view of the operator; and this arrangement should be made out of sight of the patient. Nothing more completely distinguishes the refined, gentlemanly, cultured surgeon from the boor, than his constant, watchful regard for the feelings of his patient, whose mind is harassed with anxiety and doubt, even though the operation may be a very simple one.

The surgeon should be courageous, and unflinchingly do what is required of him, but the one who has no compassion and no word of kindness for the unfortunates compelled to seek his aid, is unworthy of, and a disgrace to, the profession.

The principle upon which an instrument is made should be borne in mind, in order to its skillful use. Every cutting surgical instrument should be looked upon as a fine saw : some with the teeth set forward, towards the point, and these cut best from point to heel, as does a razor ; but the greater number are set in the opposite direction—for example, the common scalpel and bistoury—and act efficiently only when drawn from heel to point. This principle is to be borne in mind when sharpening an instrument, which most of us, living away from great centres, are obliged to do for ourselves.

The points of forceps and scissors should be kept oiled and in good easy working condition. All instruments, after use, should be made perfectly dry, and when liable to be seldom used, should be rubbed with a flannel dampened with kerosene before being put away. In operations where no anæsthetic is used, we should remember that less pain is produced when cutting from within outwards, than in a contrary direction ; hence a scalpel should be held at right angles to the part to be cut and made to penetrate directly to the desired depth, and so held while the incision is being carried to the required length that the deepest part of the incision is first made. In opening a boil or carbuncle, it hurts less to thrust a bistoury through the base and cut out than to cut down from the apex ; so in closing a wound with sutures, the thread should have a needle at either end and be introduced and drawn through from within outward. When anæsthetics are used and danger threatens therefrom, the prevailing opinion is, that it is due to a paralyzed condition of the brain, and the rational treatment is to supply the brain with blood as rapidly as possible, which is to be done by holding the patient up with head hanging down. If this fails to restore vitality in a moment or two, proceed immediately to artificial respiration.

In dressing scalp wounds take time enough to clean the wound completely, being careful to see that no hairs are left between the lips of the wound. When possible, draw the edges together by braiding the hair or tying it together over the wound. Sutures are to be avoided if not imperatively demanded in scalp wounds, because sometimes the extra irritation they produce is sufficient to develop erysipelatous inflammation. In removing encysted tumors from beneath the scalp, it is not necessary to dissect them out. If a sufficiently long incision is made over the tumor and completely down to it, being careful not to cut the cyst wall, the tumor can be easily squeezed out with the thumbs.

In cases of fracture of the skull, and especially if the fracture is of the external table only, the most efficient instrument in raising the depressed portion, in my hands, has been a common sewing awl, such as may be found in the possession of any shoemaker.

I know of no better instrument for removing particles of steel or iron from the eye than a match, with the end sharpened a little. The foreign particle adheres to it better and less injury is inflicted upon surrounding parts than when the point of a lancet is used. When particles of dirt adhere to the upper lid, the drawing of the lid well down over the under lid and holding it for a moment or two enables the winkers of the lower lid to wipe away the offending particle.

In any operation within the nostrils, or in removing of foreign substances, great aid is afforded by hooks made by bending the ends of a couple of rubber hair pins and fastening the pins to a piece of elastic long enough with a little stretching to go around the head ; then hooking the bent ends of one pin in one nostril, carrying the rubber around the head and hooking ends of the other pin in opposite nostril, the calibre of each nostril is enlarged in every direction and so held without the aid of any assistant.

In removing such substances as a kernel of corn or cherry stone which children are very apt to thrust into the nose or external meatus of the ear, and which are very apt to slip away from the grasp of forceps, the dipping the end of a pencil in solution of gum arabic or glue and holding it gently for a moment in contact with the substance, when sufficient adhesion will have taken place so that it can be withdrawn, or in the event of this experiment proving a failure, one or two horse hairs bent upon themselves in loops may be passed over the substance and by a little manipulation made to catch on the back side, when it may very readily be withdrawn.

The removal of a splinter from the finger or elsewhere, can frequently be accomplished by pressing firmly over it in the direction of its entrance with a small tube; the barrel of an ordinary watch key answers very well when the splinter is small. The pressure all around it forces the splinter up into the tube.

In dislocations of the phalanges if the phalange is bent back to an acute angle and pressure made with the thumbs on base of phalange, and at the same time with forefingers on end of bone from which it is dislocated, its reduction is generally easily effected, whereas by ordinary traction great force is required. Frequently, a dislocation of the shoulder into the axilla may be easily reduced, when other means have failed, by seating your patient on the floor with his back against a bed, then getting on the bed, taking hold of his hand and putting one foot on top of shoulder, pulling the hand and arm straight upwards.

In dislocation of the acromial end of the clavicle, which is often very difficult to keep in place by ordinary methods, I have succeeded by making a loop of adhesive plaster on the back, coming nearly up to top of shoulder, and a corresponding one on breast in front, then springing a piece of whalebone from one loop to the other over a compress on seat of dislocation and allowing it to remain until adhesions take place.

Inguinal hernia may sometimes be reduced when ordinary taxis fails, by pushing the thumb forcibly through the inguinal canal by the side of the descended intestine. I have been informed Dr. Hill once read a paper before this Society on this subject.

Nothing in my hands gives so prompt and effectual relief in orchitis, as painting the whole scrotum with a *strong* solution of nitrate of silver.

In those hard hemorrhoidal tumors found on the very verge of the anus there is no danger to be apprehended from the use of the lancet. To slit them open freely and turn out the clot is safe and very efficacious treatment. In fissures within the rectum, thorough scarification through the mucous membrane at right angles to the fissure, is pretty sure to result in cure.

In operations on the rectum, in the female, the rectum may be inverted by introducing finger into the vagina and pressing downwards and backwards, forcing it out through anus. In one case of hemorrhoidal tumors where there was a constant disposition to protrusion, great relief was found from the use of the hard rubber "pile supporter," which in this case was worn without the slightest inconvenience.

In prolapsus ani, great relief is afforded by a strip of adhesive plaster between the nates over the anus and extending an inch or more, front and back.

In chilblains, much and often permanent relief is found from painting the foot with a strong solution of carbolic acid in glycerine.

In cases of needle, glass or other substances in sole of foot, the application of Esmarch's bandage so as to completely blanch the foot, enables one easily to cut down upon and find the offending substance.

In ingrowing toe-nail the introduction under corner of nail of a small piece of compressed sponge (sponge tent) about the size of a grain of wheat gradually and without much pain, lifts the nail out of its unpleasant position, and the occasional introduction of a fresh piece perfects the cure.

In glandular and other swellings, I have never been able to find a better discutient than strong tincture of camphor. In my hands it has proved more effectual than iodine.

The combined wisdom of the ages has failed to discover any better dressing for ordinary wounds than pure water. When it is convenient to do it, the keeping of a wounded part in as hot water as can be borne, very much facilitates a cure and comes near to furnishing immunity from pain. It is necessary, however, that the water be kept all the time at a high temperature, which generally in ordinary practice it is almost impossible to do.

We are frequently consulted in cases of children unable to retain their urine at night. When remedies directed to a removal of the cause fail to produce the desired result in boys, a drop of collodion on end of prepuce furnishes protection to the bed until picked off.

Rubber bands for splints.

The tendency of the times is to a greater conservatism in surgery as in everything else. It is coming to be more generally recognized that it is more the province of the surgeon to cure than to cut. A part should never be sacrificed except when the demand for such sacrifice is imperative. To save a part which formerly would have been unhesitatingly removed, is a triumph of advancing surgical civilization, and one which, I am most happy to be able to say, is not unfrequently witnessed.

